# Identification of Gene Expression Changes from Colitis to CRC in the Mouse CAC Model

**DOI:** 10.1371/journal.pone.0095347

**Published:** 2014-04-17

**Authors:** Xin Li, Yuyan Gao, Ming Yang, Qi Zhao, Guangyu Wang, Yan mei Yang, Yue Yang, Hui Liu, Yanqiao Zhang

**Affiliations:** 1 Department of Gastrointestinal Medical Oncology, The Third Affiliated Hospital of Harbin Medical University, Harbin, Heilongjiang, China; 2 Department of Respiratory Medical Oncology, The Third Affiliated Hospital of Harbin Medical University, Harbin, Heilongjiang, China; 3 Department of Radiation Oncology, The Third Affiliated Hospital of Harbin Medical University, Harbin, Heilongjiang, China; 4 Cancer Research Institute, Harbin Medical University, Harbin, Heilongjiang, China; 5 School of Life Science and Technology, State Key Laboratory of Urban Water Resource and Environment, Harbin Institute of Technology, Harbin, China; French National Centre for Scientific Research, France

## Abstract

A connection between colorectal carcinogenesis and inflammation is well known, but the underlying molecular mechanisms have not been elucidated. Chemically induced colitis-associated cancer (CAC) is an outstanding mouse model for studying the link between inflammation and cancer. Additionally, the CAC model is used for examining novel diagnostic, prognostic, and predictive markers for use in clinical practice. Here, a CAC model was established in less than 100 days using azoxymethane (AOM) with dextran sulfate sodium salt (DSS) in BALB/c mice. We examined the mRNA expression profiles of three groups: control untreated mice (K), DSS-induced chronic colitis mice (D), and AOM/DSS-induced CAC (AD) mice. We identified 6301 differentially expressed genes (DEGs) among the three groups, including 93 persistently upregulated genes and 139 persistently downregulated genes. Gene Ontology (GO) and Kyoto Encyclopedia of Genes and Genomes (KEGG) pathway analyses revealed that the most persistent DEGs were significantly enriched in metabolic or inflammatory components in the tumor microenvironment. Furthermore, several associated DEGs were identified as potential DEGs by protein-protein interaction (PPI) network analysis. We selected 14 key genes from the DEGs and potential DEGs for further quantitative real-time PCR (qPCR) verification. Six persistently upregulated, 3 persistently downregulated DEGs, and the other 3 genes showed results consistent with the microarray data. We demonstrated the regulation of 12 key genes specifically involved in Wnt signaling, cytokine and cytokine receptor interactions, homeostasis, and tumor-associated metabolism during colitis-associated CRC. Our results suggest that a close relationship between metabolic and inflammatory mediators of the tumor microenvironment is present in CAC.

## Introduction

Colorectal cancer (CRC) is a major health problem worldwide. CRC develops through a multistage process involving the accumulation of genetic and epigenetic alterations [1]. Patients with inflammatory bowel disease (IBD) are at a higher risk for developing CRC than the general population. Several lines of evidence point to chronic inflammation of the colon as an important factor in the progression to CRC (particularly colitis-associated cancer [CAC]) in IBD [2]. Although inflammation-linked carcinogenesis is a well-accepted concept, the underlying mechanisms have not been elucidated. Inflammation can provide tumor-initiating and tumor-promoting stimuli, along with mediators that generate a tumor-prone microenvironment [3], [4], [5].

Chronic inflammation in the intestine leads to damage of the epithelium. Locally produced cytokines cause inflammation and stimulate the proliferation of crypt cells to compensate for the loss of epithelial cells. This chronically stimulated state of the epithelium may eventually lead to the development of CAC [6], [7], [8]. Therefore, targeting inflammatory mediators (i.e., chemokines and cytokines) and key transcription factors decreases the incidence and spread of cancer. Cancer-related inflammation (CRI) is the seventh hallmark of cancer [4]. However, approaches that involve targeting CRI in a therapeutic or preventative setting are in their infancy. Understanding the molecular pathways involved in CRI could contribute to revealing the underlying mechanism of inflammation-related colon carcinogenesis and could permit the development of synergistic therapies that target the inflammatory components of the tumor microenvironment. Such an approach could result in the identification of new target molecules to improve diagnosis and treatment [9].

Human studies are limited by many ethical and practical considerations [10], including i) the need for repeated surgical or endoscopic procedures to monitor the disease; ii) difficulties in controlling variables, such as individual genetic variation, environment, and diet; and iii) difficulties in studying the early steps of disease development.

Recent CRC studies in mice have focused on drug intervention in transgenic and gene knockdown animals [11]. We chose to study a BALB/c mouse model because it provides a relatively homogeneous genetic background and allows for the control of environmental factors and the application of a standard, randomized experimental design. The azoxymethane/dextran sulfate sodium salt (AOM/DSS)-induced CAC model is the most widely used model for studying colon carcinogenesis and is a reliable and practical tool [12]. However, high-throughput microarray analysis has rarely been used to assess mouse models. To expedite the identification of relevant genes that play roles in the initiation and perpetuation of experimental CAC, we applied gene microarray technology to compare gene expression patterns of experimental and control colons in a well-known mouse model of CAC. To validate the DNA microarray results, we then selected genes to investigate using real-time PCR. The aim of this study was to demonstrate that high-throughput detection techniques could be used to investigate the molecular processes that are similar in humans and animal models. To determine whether the changes of the mouse gene expression profile in the AOM/DSS model reflect what is observed in human disease, we examined novel candidate genes and revealed CAC-specific patterns in gene expression using this powerful technology.

This study has provided evidence of increased inflammation within early colon adenocarcinomas that may allow the identification of new potential pathways regulating the initiation and promotion of early colon carcinogenesis. In addition, we identified 7 novel, persistently differentially expressed genes (DEGs) and specific novel functional clusters or pathways coupled to oncogenic pathways that could be new clinical candidate markers in the process of human CAC.

Accessing the tumor microenvironment and the associated lesions is central to understanding both early colorectal carcinogenesis and the mechanisms involved in the transition of colitis to CAC. Thus, the aim of this study was to define the inflammatory microenvironment within the AOM/DSS-induced mouse CAC model and to investigate how the changes are similar to early human submucosal colonic carcinoma. Additionally, we investigated the significant gene expression changes that occur during the progression of chronic colitis to CAC.

## Materials and Methods

### Ethics Statement

Our study was approved by the Animal Care and Use Committee of the Cancer Research Institute at Harbin Medical University. All mouse procedures were performed in accordance with the Guide on the Ethical Use of Animals of the Cancer Research Institute at Harbin Medical University, and all efforts were made to minimize suffering. The protocol was approved by the Committee on the Ethical Use of Animals of the Cancer Research Institute at Harbin Medical University (Permit Number: 2012-003).

### Establishment of the Colitis and Colitis-associated Cancer Models

We followed a published protocol [13], [14] to establish mouse models using two drugs: AOM and DSS [15], [16]. Studies were performed with 48 female BALB/c mice (5–7 weeks old) provided by the Cancer Research Institute of Harbin Medical University. All mice were maintained at room temperature (20–22°C) with humidity (40–60%) and 12-h light/dark cycles. The animals were supplied a pelleted basal diet and drinking water. They were quarantined for the first 7 days with regular diet and water intake and were then randomized by body weight into experimental and control groups (n = 12/each group). In brief, chronic colitis was induced by 3 cycles of 2.5% DSS in drinking water for 5 days followed by drinking distilled water for 16 days. Colitis-associated cancer was induced by intraperitoneal injection of a single dose of the mutagenic agent AOM (12.5 mg/kg, Sigma Aldrich, St. Louis, MO) on day 1 followed by 3 cycles of 2.5% DSS (MP Biomedicals, Santa Ana, CA) in the drinking water for 5 days and then distilled water for 16 days. The animals in the control group were untreated. All mice were sacrificed at the end of the study (week 14, 100 days). The experimental procedure is shown in Figure S1.

### Tissue Processing, Collection, and Histopathologic Evaluation

After sacrifice, the distal colon was removed (a 4-cm segment of the left colon), where CRC and colitis frequently occur in this model. The tissue samples were scored for macroscopic damage using an established defined scoring system [17], [18].

Distal colonic epithelial tissue samples from the AOM/DSS, DSS, and control groups were harvested as described above. Then, one-half of each sample was scraped using a slide, immersed in TRIzol reagent (Invitrogen, Carlsbad, CA), and stored frozen in liquid nitrogen at −80°C, while the other half (adjacent to the sampling site) was fixed in buffered formalin for histological and immunohistochemical analysis as described previously [19]. The neoplasm samples from the AOM/DSS group were clipped using ophthalmic scissors and kept in RNA storage solution (Tiangen, Peking, China).

### Genome-wide Gene Expression Profile Analysis

Total RNA was extracted, and mRNA expression was measured using the Whole Mouse Genome Microarray Kit, 4x44K (Agilent Technologies, Santa Clara, CA), which contained 41,174 oligonucleotide probes representing more than 41,000 mouse genes and transcripts. Three samples from each group were used to detect mRNA expression, a process that was completed by Shanghai Biochip Co., Ltd (Shanghai, China), according to the manufacturer’s instructions.

GeneChips were scanned using an Agilent Microarray Scanner (Cat#G2565CA, Agilent technologies, Santa Clara, CA, US) with default settings – Dye channel: Green, Scan resolution = 5 µm, PMT 100%, 10%, 16 bit. The data were analyzed using Feature Extraction software 10.7 (Agilent technologies, Santa Clara, CA, US) and were normalized with the Quantile algorithm in Gene Spring Software 11.0 (Agilent technologies, Santa Clara, CA, US). All microarray data, along with the design parameters, have been deposited in NCBI’s Gene Expression Omnibus (GEO) and are accessible through GEO Series accession number GSE44988 (http://www.ncbi.nlm.nih.gov/geo/query/acc.cgi?acc=GSE44988).

### Identification of Significant Differentially Expressed Genes

The differentially expressed genes among the AOM/DSS (AD), DSS (D), and control (K) sets were identified using the R statistical software package (www.r-project.org). The significant differentially expressed genes were defined as the genes with fold changes of >2 or <0.5 and with adjusted p values <0.05. The differentially expressed genes with fold changes between 2 and 0.5 were removed from subsequent analysis. The significant differentially expressed genes between AD and K, D and K, and AD and D were identified. In the comparisons of D vs. K, AD vs. K, and AD vs. D, the genes that were all upregulated in the three comparisons were identified as the persistently upregulated genes, and the genes that were all downregulated in the three-way comparisons were defined as the persistently downregulated genes.

### Enrichment Analysis of the GO and KEGG Pathways

The gene annotation enrichment analysis using Gene Ontology (GO) (http://www.geneontology.org/) and Kyoto Encyclopedia of Genes and Genomes (KEGG) (http://www.genome.jp/kegg/) data for gene sets was performed using DAVID software [20]. This software can provide a functional interpretation of large gene lists derived from genomic studies. A Benjamini p-value of <0.05 was used in the analysis.

### Clustering Analysis

The unsupervised hierarchical clustering of the mouse expression profiles of the AD, D, and K sets was performed using Cluster and TreeView (http://rana.lbl.gov/EisenSoftware.htm). The functional categorization using DAVID was based on the GO of the sum of the differentially expressed genes clustered using CIMminer and the Euclidean distance [21].

### Mouse Protein-protein Interaction Network Construction

The mouse protein-protein interaction data were obtained from MppDB, which is a mouse protein-protein interaction (PPI) database [22]. The reference set was used for the construction of the background network, which was collected from five PPI databases; DIP [23], BIND [24], MIPS [25], MINT [26], and IntAct [27]. The DEG-associated subnetwork is composed of the DEGs and the genes that are connected with these genes in the background network.

### Network Visualization and Module Detection

The networks were drawn using Cytoscape (http://www.cytoscape.org/), an open-source software platform for visualizing complex biological networks. Modules of the subnetwork were detected using MCODE, which is available as a plug-in for the Cytoscape network visualization software [28].

### Validation of Microarray Data by Quantitative Real-time PCR (Real-time PCR)

Total RNA was isolated from colonic mucosal biopsy samples with TRIzol reagent or RNA storage solution. This study analyzed control colon tissue (n = 3), an AOM plus DSS-treated group (n = 3), and a DSS induced-chronic colitis group (n = 3).

Total RNA was reverse-transcribed using oligo(dT) primers (Takara). Real-time PCR was performed using the ABI 7500 Real-Time PCR system (Applied BioSystems, Carlsbad, CA, USA). The primers for 14 selected genes (listed in Table S1) were used for validating of microarray data. The PCR cycling conditions were 95°C for 10 minutes followed by 40 cycles at 95°C for 15 seconds and template extension for 30 seconds at 72°C. *Gapdh* expression was used as an internal control. The expression levels of the following genes were analyzed: *Asprv1*, *Slc16a10*, *Pacsin3, Sycn, 0610005c13Rik*, *Tnfrsf11b, Inhbb*, *Cxcl5*, *Cxcr2*, *Cxcr5*, *Prkcz*, *Tnfsf9*, *Orc2*, and *Orc5*.

## Results

### Analysis Based on the Microarray Data

The carcinogenic effects of AOM combined with DSS administration have been verified in a previous study. CAC develops more frequently in the AOM/DSS-treated female BALB/C mice by day 100 (14–15 weeks) [12]. In this study, we established and successfully evaluated the histopathology of an AOM/DSS-induced mouse CAC model. We observed a few spotted mucosal ulcers, submucosal carcinomas with regenerative changes, and/or chronic inflammation in the AOM/DSS and DSS groups but not in the untreated groups (as described in the Materials and Methods). We then isolated total RNA from mucosal samples from K, AD, and D mice taken at autopsy. The RNA samples were then evaluated using microarray analysis.

**Figure 1:**
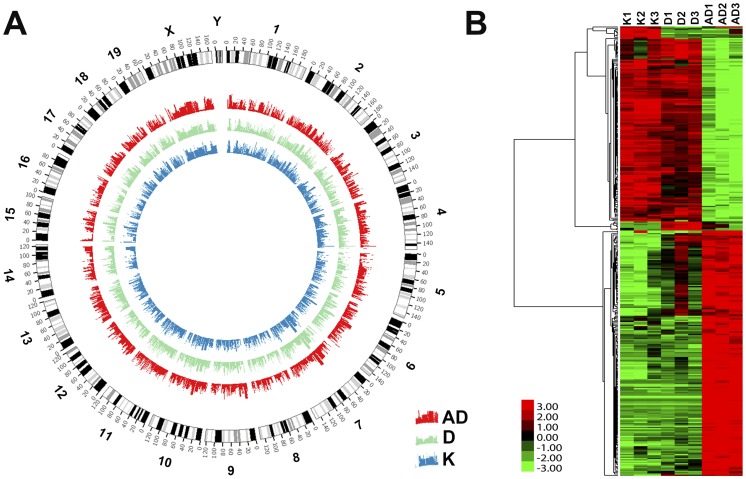
The gene expression profiles and the differentially expressed genes of the three states. (**A**) Comparison of mouse gene expression profiles in the three states plotted in Circos format. Red represents the AD group, green represents the D group, and blue represents the K group. The positions of the genes were matched into mouse genome assembly mm9. (**B**) Unsupervised hierarchical clustering of 6301 DEGs of the K, D, and AD groups.

The mouse expression profiles were scanned using an Agilent Whole Mouse Genome Oligo Microarray (4×44K) platform for the states of AD, D, and K, and each state was assessed with 3 biological replicates. First, we analyzed the biological replicates among the three groups (AD, D, and K) and the correlations of the samples in each state. The findings indicated that the replicates were highly positively correlated and that the biological samples had significant repeatability in the same state (Pearson’s correlation coefficient, Figure S2). Analysis of data in Circos format revealed significant gene expression differences among the groups (Figure 1A). The positions of the genes were matched against mouse genome assembly mm9. Figure 1A shows that there were significant differences in the expression profiles among the three groups (AD, D, and K) within the whole genome. We then compared the expression profile data among the three states and identified the significant DEGs for each condition (D vs. K, AD vs. K, and AD vs. D). To evaluate whether the gene expression changes were sufficient to distinguish the three different states, we performed unsupervised hierarchical clustering for these DEGs in the three states (K, D, and AD) Figure 1B shows that there were significant differences for the three states and that the expressive characteristics of the data were consistent within each group (AD, D, and K). Some genes were differentially expressed in only one or two comparisons, and some genes were persistently dysregulated in all three comparisons. These results indicated that mouse chronic colitis and inflammation-associated colon cancer specimens have their own characteristic genetic profiles.

### Analysis of Mouse DEGs in the Colonic Mucosa

**Figure 2:**
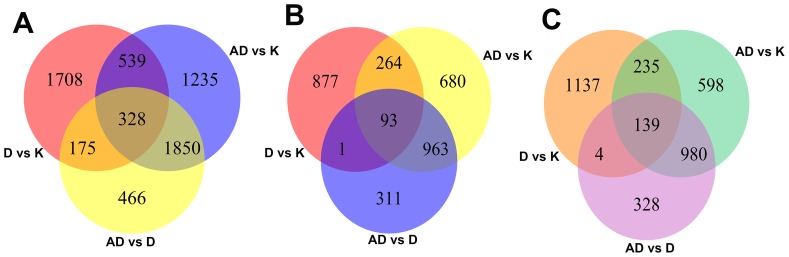
The number counts of the differentially expressed genes. (A) Venn diagram showing the number of unique and overlapping DEGs. Separate studies on up/downregulated DEGs are described in (B) and (C), respectively.

To investigate the disease-related processes, we compared the expression profile data for each two-state comparison and identified the significant DEGs in each comparison (D vs. K, AD vs. K, and AD vs. D) using the R statistical package (samr). Probe sets with fold changes ≥2 or ≤0.5 with adjusted p values <0.05 were chosen as DEGs for further analysis. Figure 2A–2C shows there are 2750, 2819, and 3952 DEGs in the D vs. K, AD vs. D, and AD vs. K comparison sets, respectively. The change in the number of differentially regulated genes was greater in the AD vs. K group than in the other two-state comparisons.

We also identified unique and overlapping DEGs in all three groups. The gene numbers are shown in Figure 2A . Moreover, there are only 328 DEGs common to all three comparisons (D vs. K, AD vs. K, and AD vs. D) (Figure 2A). Some important information for these 328 overlapped DEGs is provided in Table S2, including p values, the mean expression level of each gene in each group, the fold changes, etc. To gain insight into disease-related processes during mouse CAC, we next focused on DEGs identified in CAC mice. As shown in Figure 2B–2C, in total, 232 genes were all differentially expressed in the three-way comparisons and were identified as the persistently dysregulated genes, including 93 that were upregulated and 139 that were downregulated. The persistent DEGs were enriched using GO and KEGG analyses.

### GO and KEGG Pathway Enrichment Analysis of DEGs in a Mouse CAC Model

To gain new insights into the dynamic molecular and signaling changes that occur during mouse CAC development, we used GO analysis of 232 statistically significant persistent DEGs. Our analysis indicated that 22 categories were enriched by DAVID and included genes associated with the following functions: cell differentiation; extracellular structure organization; localization; lipid metabolic processes; regulation of cell adhesion; cell motion; cellular, organ and system development processes; etc. (Table 1). These findings suggested that the dysregulation of these biological processes might link colitis to CRC.

**Table 1:** GO enrichment analysis of persistent DEGs from K to D to AD.

Go name	gene num	P value
cell differentiation	40	6.48E-03
cellular developmental process	45	7.65E-03
extracellular structure organization	8	1.22E-02
positive regulation of biological process	37	1.39E-02
response to cadmium ion	3	1.62E-02
regulation of cell adhesion	6	2.01E-02
anatomical structure development	49	2.09E-02
myeloid cell differentiation	6	2.18E-02
lipid metabolic process	0	2.27E-02
system development	46	2.39E-02
hematopoiesis	10	2.46E-02
organ development	39	2.52E-02
sensory organ development	10	2.80E-02
regulation of cell motion	6	3.28E-02
regulation of epidermis development	3	3.52E-02
alcohol metabolic process	12	3.87E-02
negative regulation of cellular process	29	3.87E-02
Localization	56	4.32E-02
hematopoietic or lymphoid organ development	10	4.52E-02
erythrocyte differentiation	4	4.52E-02
skeletal system development	10	4.86E-02
cellular process	167	4.88E-02

**Figure 3:**
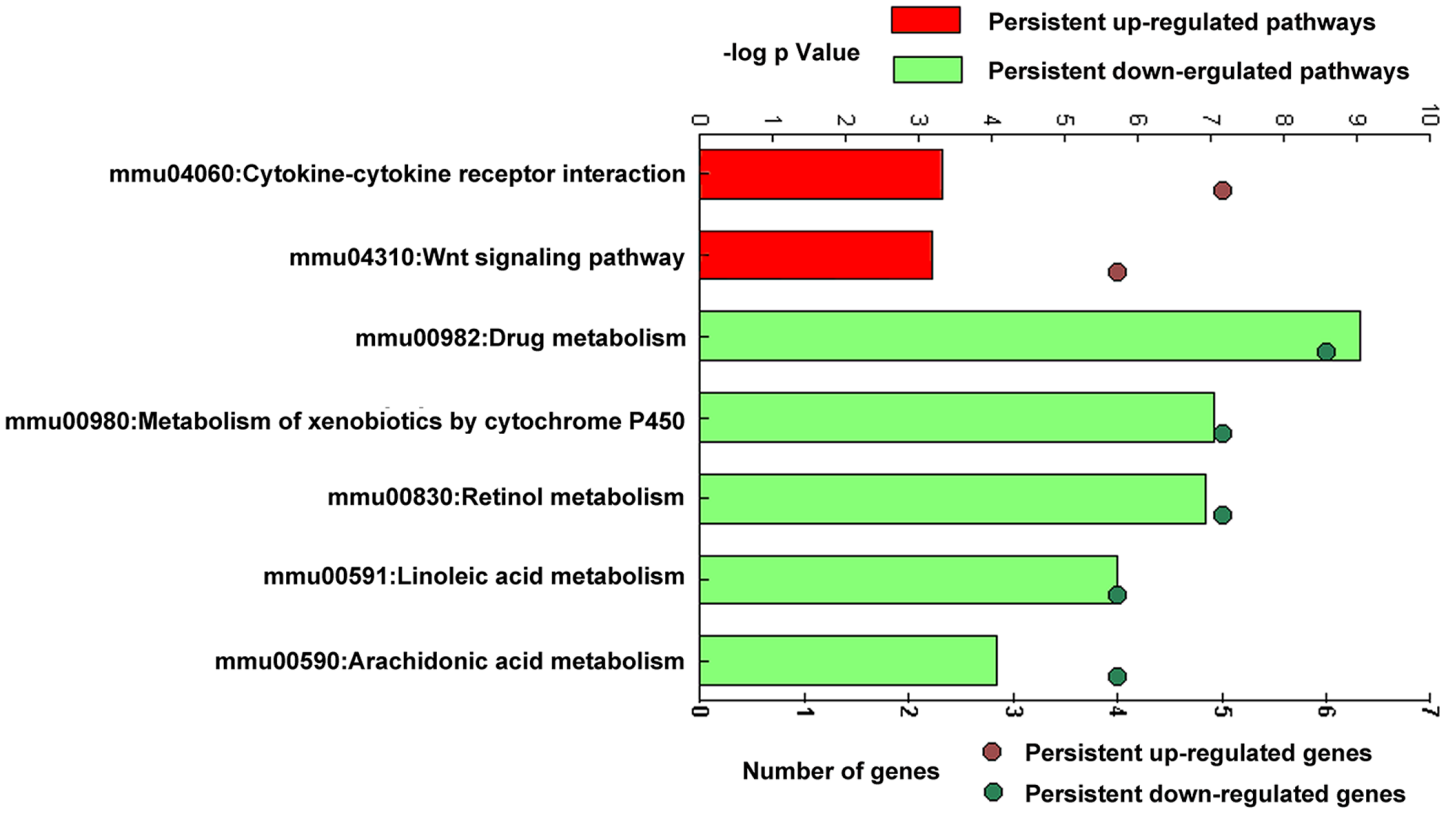
Pathway analysis of persistent DEGs among the K, D, and AD groups. Red bars represent the persistently upregulated KEGG pathway enrichment significance levels, and green bars represent the persistently downregulated KEGG pathway enrichment significance levels (upper axis). The triangles denote the number of genes differentially expressed in the upregulated pathways, and circles denote the number of genes differentially expressed in the downregulated pathways (lower axis).

A total of 232 persistent DEGs from K to D to AD were analyzed for KEGG pathway enrichment. Seven significantly enriched pathways were identified: 2 pathways were persistently upregulated, and 5 were persistently downregulated. The most enriched pathways are shown in Figure 3. A detailed analysis of upregulated pathways revealed the activation of both cytokine-cytokine receptor interactions and the Wnt signaling pathway. Both cytokine receptors and Wnt signaling are involved in the development and progression of cancers. Along with being involved in cancer-related pathways, members of the cytokine-cytokine receptor interaction pathways are also crucial intercellular regulators and mobilizers of cells engaged in innate and adaptive inflammatory host defenses, cell growth, differentiation, cell death, angiogenesis, and development and repair processes of homeostasis. The five downregulated pathways are involved in tumor regulation of metabolism, including metabolism of xenobiotics by cytochrome P450, retinol metabolism, and arachidonic acid metabolism. These pathways are similar to the enriched dysregulated pathways for AD versus D.

### Predicting Cancer-related Genes Using a PPI Network

To screen potential biomarkers of inflammation and colon cancer transformation, we used a protein-protein interaction (PPI) network for further analysis. In this study, we used a PPI network as a background network to understand the differentially expressed gene patterns present in the development and progression of cancers. The background network, the mouse protein-protein interaction network, was obtained from MppDB. This background network contained 5136 interaction pairs covering 10,337 mouse genes (Figure S3).

In total, 6301 DEGs were used as seeds and were mapped to the background network, and a subset of 793 genes was obtained. Using this subset, a DEG-associated subnetwork was extracted from the background network. The subnetwork contains the DEGs and the genes that connect with the differentially expressed genes in the background and consists of 5193 genes (nodes) and 2863 interaction pairs (linkages) (Figure S4).

**Figure 4:**
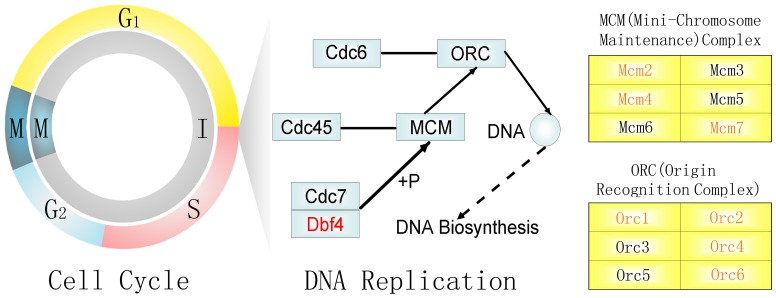
Model of the annotated pathways for module 1. The red genes are the members of this module, and Mcm4 and Mcm7 are the DEGs.

**Figure 5:**
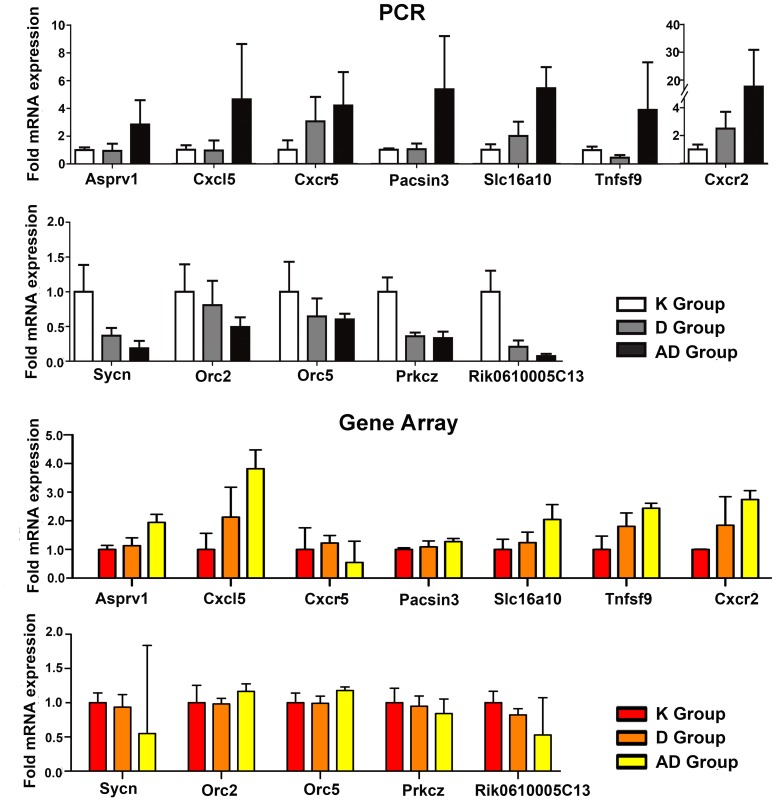
Gene expression levels of 12 selected genes in the three groups (K, D, and AD). The above panel shows the gene expression levels as detected by real-time PCR, and the bottom panel shows the gene array data.

This analysis used MCODE to identify 24 modules of the subnetwork. The first module had the highest score according to the densest clique, contained 9 nodes and 32 edges (Figure S5)**.** In the module, 8 genes were annotated in DNA replication and the cell cycle by KEGG, including the two differentially expressed genes. Two nodes, named *Mcm4* and *Mcm7*, were upregulated in AD vs. K and in AD vs. D. These findings suggested that any gene expression level changes in module 1 (Figure 4) could result in the dysregulation of cell cycle progression and could affect the progression of inflammation and cancer (Figure 4). A complex network of DEGs and their associated interacting proteins and enzymes will be required to investigate novel biomarkers associated with mouse CAC. Some DEGs (*Pacsin3* and *Prkcz*) and DEG-associated interacting proteins and enzymes (*Orc2,* and *Orc5*) have been validated by real-time PCR (Figure 5).

### Real-time PCR Validation of Microarray Data

To validate the microarray results, we conducted real-time PCR to quantify the expression levels of 14 key genes from the microarray and PPI, including 6 genes (*Cxcl5, Cxcr2, Tnfsf9, Tnfrsf11b, Cxcr5, and Inhbb*) associated with CRC progression and enriched in the cytokine-cytokine receptor interactions pathway, 6 novel DEGs associated with epithelial damage repair remodeling and metabolic regulation, and 2 potential DEGs (*Orc2, and Orc5*) involved in the regulation of cell cycle and DNA replication. Among 14 key genes, on the basis of fold changes of >2 or <0.5 and with adjusted p values <0.05, we selected 12 DEGs for real-time PCR verification, including 6 persistently upregulated DEGs (*Asprv1, Slc16a10, Pacsin3, Cxcl5, Cxcr2, and Tnfsf9),* 3 persistently downregulated DEGs (*Sycn, 0610005c13Rik, and Prkcz*), and the other 3 genes (*Inhbb, Tnfrsf11b, and Cxcr5*). Based on the results of real-time PCR results, we demonstrated that 6 genes (*Asprv1, Slc16a10, Pacsin3, Cxcl5, Cxcr2, and Cxcr5)* were persistently upregulated and 3 genes (*Sycn, 0610005c13Rik, and Prkcz*) were persistently downregulated. The results indicated that the expression levels of targeted genes (9/12, 75%) were consistent with the microarray data (Figure 5). The genes *Cxcr5, Inhbb, and Tnfrsf11b* were exceptions, as significant changes in the three genes were not demonstrated in the microarray data, although significantly decreased (*Cxcr5*) and increased (*Inhbb, and Tnfrsf11b*) levels were observed in the DSS and AOM/DSS groups, respectively. This difference was likely due to the increased sensitivity of real-time PCR compared with high-throughput microarray analysis. High *Inhbb and Tnfrsf11b* expression, low *Cxcr5* expression are consistent with our understanding of the three genes’ function in tumor microenviroment. *Orc2* and *Orc5* gene from the PPI analysis did not show significant changes in expression level according to the microarray data, but they were selected for validation as they are interacting proteins and enzymes associated with DEGs (*Mcm4* and or *Mcm7*) and play key roles in regulating the cell cycle and DNA replication. Additionally, two genes (*Orc2* and *Orc5)* show persisitently significant downregulated levels from K to D to AD, which have been validated by real-time PCR (Figure 5). Thus, the potential DEGs (*Orc2* and *Orc5)* can be used as new and meaningful molecular markers in the evolution process of chronic colitis to colitis-associated CRC in this model.

## Discussion

Chronic inflammation is a well-known risk factor for colon cancer. Although it has been widely accepted that inflammation plays a critical role in colorectal cancer initiation, promotion, and progression, the mechanisms underlying CAC development are not clear. The identification of biomarkers of diagnosis, treatment, and prognosis is an unmet clinical need that requires urgent attention. In this study, we established an AOM/DSS-induced mouse CAC model that has been widely used to elucidate the mechanisms and to test novel therapeutic strategies for CAC, especially in the earlier stages [11]. AOM is a major CRC-inducing agent that causes O6-methylguanine formation in rodents [15]. DSS has been used in repeated administrations to induce chronic colitis [16]. This model exhibits phenotypic and genotypic features similar to those observed in human CAC. In addition, the identification of new target molecules, which could lead to improved diagnosis and treatment in CAC patients, is critical for future investigations.

In this study, we examined the mRNA expression profiles of colonic tissues to demonstrate inflammation and carcinoma at 100 days in the AOM/DSS model. Similar to a recent study [29], our results identified differentially expressed genes associated with apoptosis, inflammatory and immune defense processes, and several cancer-related pathways. We compared three differential states (AD vs. D, D vs. K, and AD vs. K) and focused on 328 DEGs (Figure 2A). Most of the 328 genes were enriched in some pathways during CAC (in the process of D vs. K →AD vs. D →AD vs. K), including Wnt signaling; cytokine-cytokine receptor interactions; homeostasis; cell differentiation; localization; tumor-associated metabolism; regulation of cell adhesion; and cellular, organ, and system developmental processes (Table 1, Figure 3). Additionally, we identified 7 categories relevant to mouse CAC progression that enriched persistent DEGs from K to D to AD. Our study confirmed that a mechanism for cytokine-cytokine receptor interaction and Wnt signaling upregulation also exists in colitis-associated CRC (Figure 3) and could activate the transcription of inflammation-related molecules. The Wnt signaling pathway was activated by 14–15 weeks in the AOM/DSS treatment group. This result is consistent with the functions of the Wnt signaling pathway, such as stimulating proliferation, maintaining stem cell characteristics, and promoting tumor development [30].

This study also identified 7 novel genes (*Asprv1, Slc16a10, Pacsin3, Sycn, 0610005c13Rik, Orc2,* and *Orc5*) that have not been previously associated with CRC. These genes could be potentially related to the pathogenesis of inflammation, IBD, and other inflammation-related cancers. The genes *Asprv1*, *Slc16a10*, and *Pacsin3* were persistently upregulated and others were persistently downregulated from K to D to AD. Interestingly, the findings showed that the 7 novel genes had more significant differences in the AD vs. K group than in the D vs. K group by real-time PCR. The results suggest that these genes can be used as new and meaningful molecular markers in the evolution process of chronic colitis to colitis-associated CRC in this model. The genes *Orc2* and *Orc5* are interacting proteins and enzymes associated with DEGs (*Mcm4* and or *Mcm7*) in module 1. Both genes play key roles in regulating the cell cycle and DNA replication, and these genes have been validated by real-time PCR (Figure 5). A recent study showed that participation of cell cycle regulators and oncogenic proteins in cancer development extend beyond the control of cell proliferation [29]. Therefore, both genes could be novel biomarkers associated with mouse CAC and have not been previously associated with human CRC. Thus, these genes should be further confirmed because they are mouse orthologs of human genes. Further analyses of the biological functions of the 7 novel genes are needed, and whether these genes are also changed in human normal colon, IBD, and CAC specimens is unclear. If these genes are differentially regulated, a new hypothesis of CRC formation could be developed. Additionally, novel functional categories that also deserve further exploration include “regulation of ion transport and homeostasis”, “ncRNA and glutamine family amino acid metabolic processes”, “stress-activated protein kinase signaling pathways”, and “regulation of lymphocyte energy”. These findings are expected to promote the development of novel hypotheses that will likely guide future research directions.

Additional studies have recently elucidated the important role of metabolism in carcinogenesis. A close relationship between cell proliferation and metabolism may occur by common regulatory pathways in cancer cells. Recently, Sharp JA et al. identified new targets (including Pacsin3) for breast cancer treatment using a cDNA microarray [31]. Furthermore, Roach W et al. observed that Pascin3 overexpression increases adipocyte glucose transport through GLUT1 [32], which is consistent with our data because *Pacsin3* is associated with negative regulation of transport. The dysregulation of glucose and lipid metabolism increases in patients with metabolic syndrome, including obesity, diabetes, and hyperlipidemia. These metabolic changes increase CRC risk, and some medicines used to treat metabolic disorders could also be used as complementary approaches to antitumor therapies [33]. Our study suggests that *Pacsin3* upregulation and the activation of metabolic pathways may also occur in CAC. Recent data from Hildenbrand M et al. demonstrated that Asprv1 (an AP-1-dependent target gene) plays a crucial role in the differentiation and homeostasis of multilayered epithelia [34], and aberrant Asprv1 expression causes impaired skin regeneration and remodeling after cutaneous injury and chemically induced hyperplasia. In this study, *Asprv1* was upregulated in DSS (2.127-fold) and AOM/DSS (62.102-fold) treatment groups. *Asprv1* is overexpressed after DSS-induced mouse colon chronic inflammation injury and AOM/DSS-induced colon hyperplasia. Based on GO analysis, this gene is associated with the development of anatomical structures, organs, and systems. Thus, we hypothesize that aberrant *Asprv1* expression causes intestinal epithelial damage repair remodeling, which results in a series of immune inflammatory responses. Because immune inflammatory responses result in abnormal differentiation and proliferation of the intestinal epithelium, the homeostasis of the intestinal barrier is destroyed, and CRC develops.

Syncollin (Sycn) is a secretory granule protein that binds to syntaxin in a calcium-sensitive manner. It is expressed in rat and human pancreas, spleen, duodenum, and colon [35], [36]. Previous research has demonstrated that Sycn was a potential pancreatic tumor biomarker for the early detection of pancreatic secretions in pancreatic cancer patients by quantitative proteomic analysis [35], [37]. Additionally, the Sycn protein is significantly increased in plasma from pancreatic cancer patients by enzyme-linked immunosorbent assay (ELISA) and integrated proteomic profiling of cell lines and pancreatic secretions [38]. Our data indicate that *Sycn* was persistently downregulated in the D (0.414-fold) and AD (0.007-fold) groups compared to the K group by real-time PCR in the chemical-induced model. In this study, distal colon tissues, where the cytokine distribution and mucus secretion of the intestinal epithelium are different from the proximal small intestine, exhibit differential gene expression. The DEGs and cancer-related pathways may represent possible diagnostic and therapeutic targets for CRC. Therefore, we evaluated 7 key novel persistent DEGs in an attempt to identify additional carcinogenic mechanisms and effective interventions for inflammation-induced CRC.

Recent studies have revealed interesting common features shared between colitis and CAC. CAC is likely the result of the stepwise activation of a complex series of molecular events that begins with tissue damage and inflammation. It is now widely accepted that animal models play a major role in elucidating mechanisms and testing novel therapeutic strategies to treat CAC [39]. We chose to study the AOM/DSS model because of its high reproducibility and ease of operation. This model has become an appropriate tool to study colon carcinogenesis [12]. We demonstrated that gene expression profiling has the potential to identify the molecular events involved in an animal model mimicking human CAC. Array profiling is a powerful tool that expedites the analysis of multiple genes simultaneously and identifies reliable clinical parameters for CAC occurrence. It is essential to identify DEG profiles in this model using mRNA microarray and to compare them with the profiles that characterize human IBD and CRC. However, unlike RNA-Seq, DNA microarrays have several limitations, including its reliance on an existing genome sequence, high background levels, requirement for a greater amount of RNA, an inability to reveal the precise location of transcription boundaries (to single-base resolution), and a limited dynamic range of detection. Therefore, combining arrays with RNA-Seq technology will be feasible for studying the complexity of the cancer transcriptome with greater efficiency and higher resolution. A combined approach will allow for the investigation of alternative splicing, isoform usage, gene fusions, and novel transcripts [40], [41], [42]. The data obtained from such studies will be analyzed using extensive computational approaches to examine the expression data and to reveal associations between CRC and inflammation.

In summary, our study provides evidence supporting the CAC mouse model as a useful tool for understanding the molecular pathways involved in CRI. In the current study, we presented a global view of molecular events happened in the process of colitis-associated carcinogenesis in a mouse model. We demonstrated the expression of metabolic genes and 7 novel key genes were persistently dysregulated during colitis-associated CRC, including the significantly dysregulation of several important cancer-related pathways and the expression of inflammatory cytokines and chemokines involved in the tumor microenvironment. Our study confirmed that a mechanism for cytokine-cytokine receptor interaction and Wnt signaling upregulation also exists in colitis-associated CRC (Figure 3) and could activate the transcription of inflammation-related molecules. Our results suggest that a close relationship between metabolic and inflammatory mediators of the tumor microenvironment is present in CAC. Furthermore, our study increases the understanding of the underlying mechanism of inflammation-mediated colon carcinogenesis. Despite the limitation that our study lacked detailed in vivo or in vitro experimental validations, our results do provide preliminary evidence for uncovering novel candidate therapeutic targets for human CAC. Our study suggests that taking advantage of specific blockage- or activation-related molecules or pathways in different stages of malignant transformation will create new insights for therapeutic and preventive methods for CAC.

## Supporting Information

Figure S1 **Experimental procedure and pathological observation of the CAC mouse model: the AOM/DSS group, the DSS group, and the control group.**
(TIF)

Figure S2 **Scatter plots and correlation coefficients for biological replicates in each state.** A, B, and C show the correlations of samples in the three states, K, D, and AD, respectively. Drep1, Drep2, and Drep3 represent the replicates for K, D, and AD, respectively.
(TIF)

Figure S3 **The mouse background PPI network.** There are 10,337 nodes (mouse genes) and 5136 linkages (protein-protein interactions) in the background network.
(TIF)

Figure S4 **The mouse DEG-associated subnetwork.** There are 5193 nodes (mouse genes) and 2863 linkages (protein-protein interactions) in the background network. The yellow nodes represent the DEGs.
(TIF)

Figure S5 **The module with the highest score in the subnetwork identified by MCODE.** The yellow nodes represent the DEGs.
(TIF)

Table S1 Primer sequences for the 12 selected genes and the housekeeping gene.
(DOC)

Table S2 **Overlapping genes from the three-way comparisons.** The first three columns represent the gene symbols, Entrez gene IDs, and gene descriptions, respectively. The fourth column indicates whether the genes are persistently dysregulated genes, representing by non-dysregulated, downregulated, and upregulated. The next three columns show the fold changes for the three-way comparisons. The last three columns represent the mean expression levels of the three states: K, D, and AD.
(XLS)
