# Supplementary figures and images for: Identification of Gene Expression Changes from Colitis to CRC in the Mouse CAC Model
Source: PLoS One. 2014 Apr 17;9(4):e95347. doi: 10.1371/journal.pone.0095347 (PMC3990644; doi:10.1371/journal.pone.0095347)

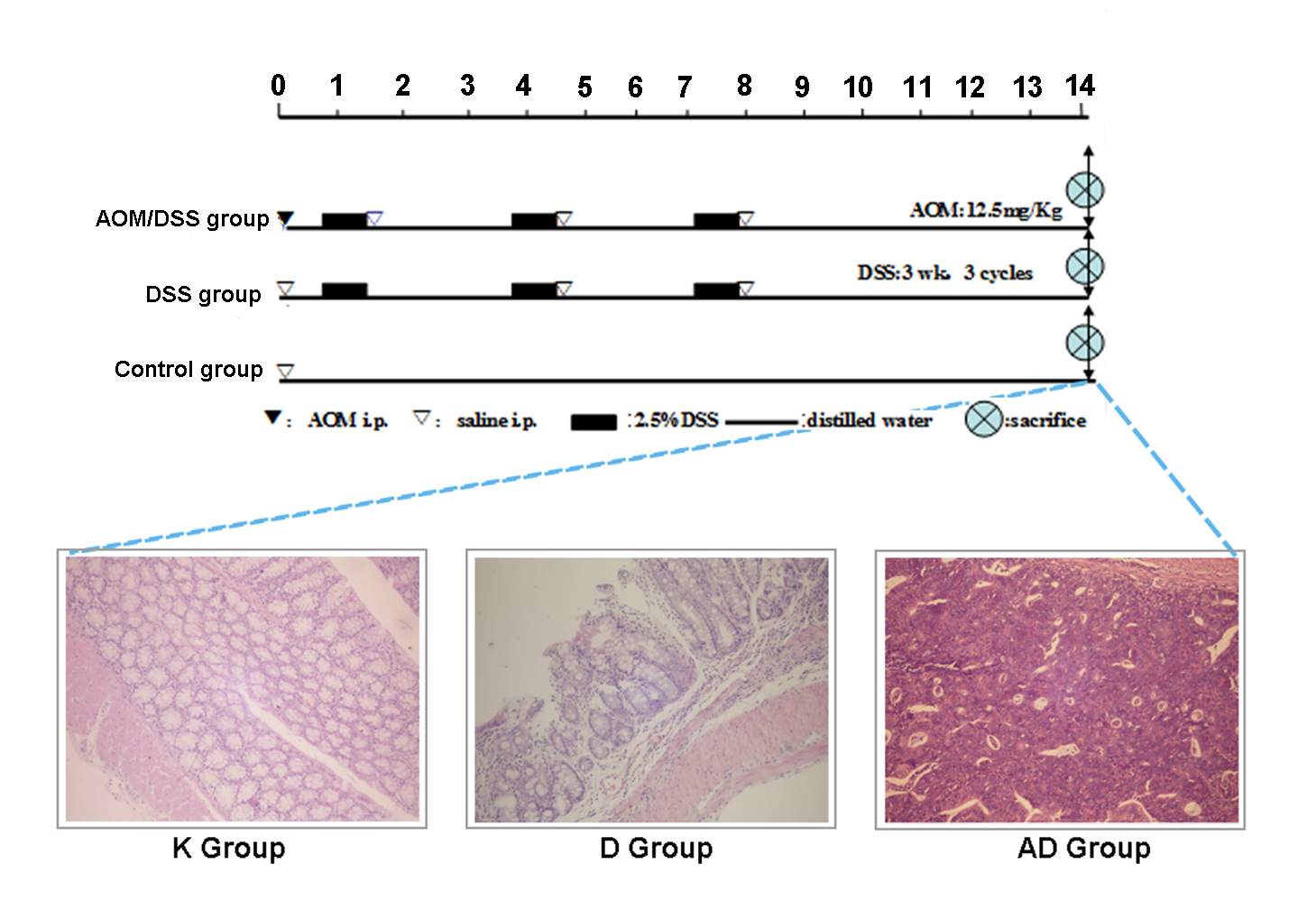

Supplement: Figure S1 — Experimental procedure and pathological observation of the CAC mouse model: the AOM/DSS group, the DSS group, and the control group. (TIF) [file pone.0095347.s001.tif]

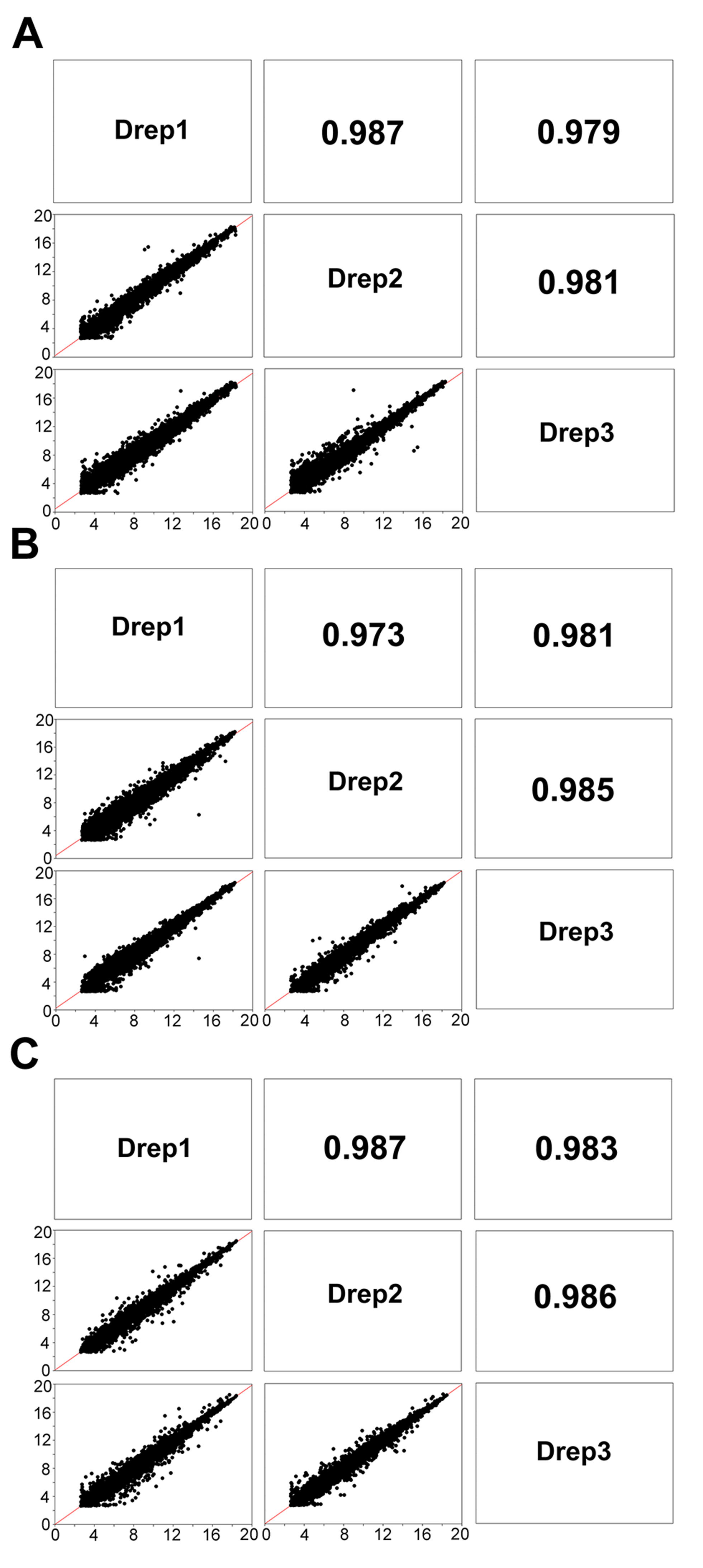

Supplement: Figure S2 — Scatter plots and correlation coefficients for biological replicates in each state. A, B, and C show the correlations of samples in the three states, K, D, and AD, respectively. Drep1, Drep2, and Drep3 represent the replicates for K, D, and AD, respectively. (TIF) [file pone.0095347.s002.tif]

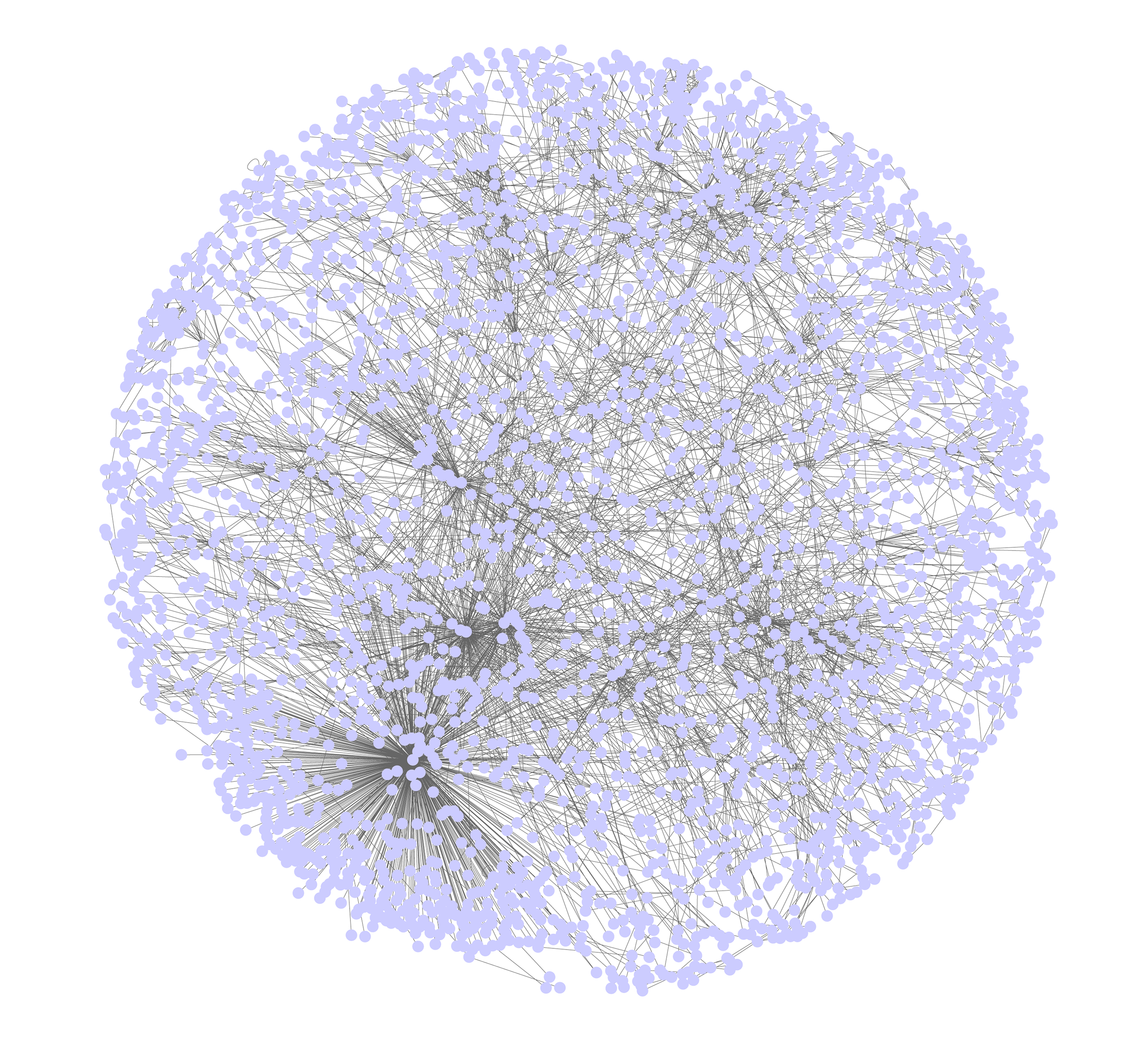

Supplement: Figure S3 — The mouse background PPI network. There are 10,337 nodes (mouse genes) and 5136 linkages (protein-protein interactions) in the background network. (TIF) [file pone.0095347.s003.tif]

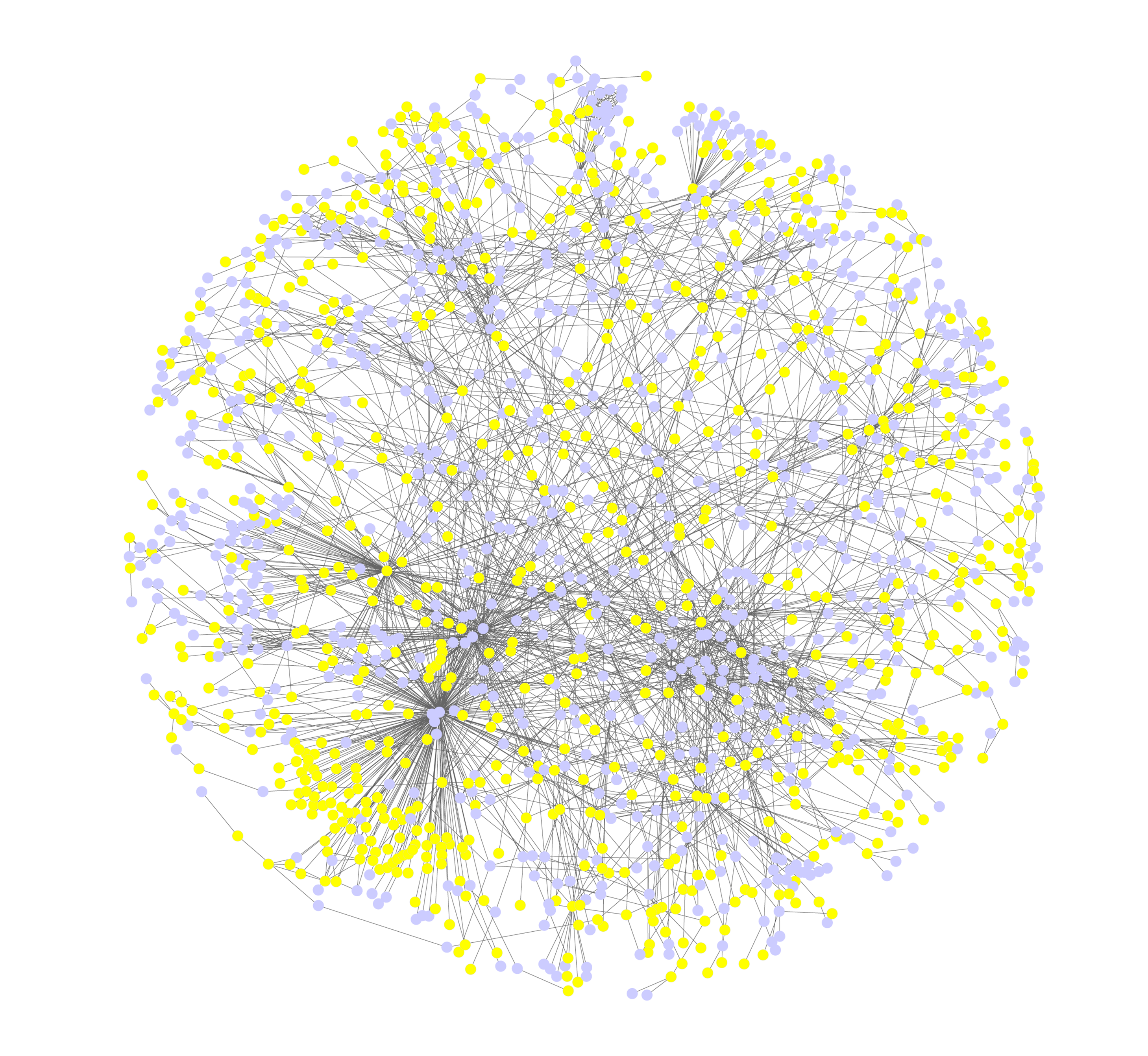

Supplement: Figure S4 — The mouse DEG-associated subnetwork. There are 5193 nodes (mouse genes) and 2863 linkages (protein-protein interactions) in the background network. The yellow nodes represent the DEGs. (TIF) [file pone.0095347.s004.tif]

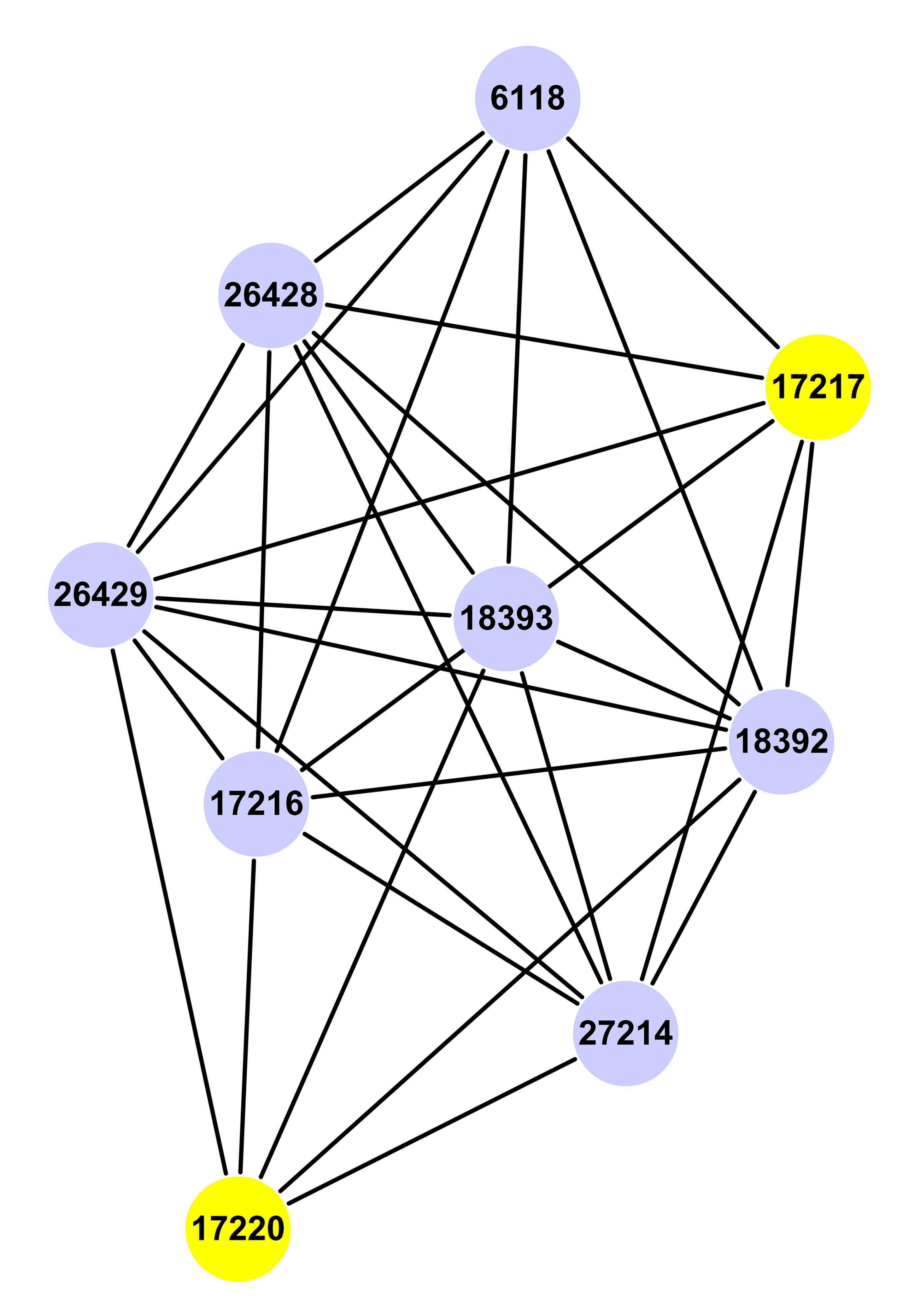

Supplement: Figure S5 — The module with the highest score in the subnetwork identified by MCODE. The yellow nodes represent the DEGs. (TIF) [file pone.0095347.s005.tif]
