# Supplementary material for: Identification of Gene Expression Changes from Colitis to CRC in the Mouse CAC Model
Source: PLoS One. 2014 Apr 17;9(4):e95347. doi: 10.1371/journal.pone.0095347 (PMC3990644; doi:10.1371/journal.pone.0095347)
Supplement: Table S1 — Primer sequences for the 12 selected genes and the housekeeping gene. (DOC) [file pone.0095347.s006.doc]

**Table S1. Primer sequence**

| Gene name | Forward sequence | Reverse sequence | Product length |
| --- | --- | --- | --- |
| *Asprv1* | 5’-CAGCCAGCCAGACACTT-3’ | 5’-GAGCGACTCTTTCAGGAAC-3’ | 342bp |
| *Slc16a10* | 5’-GAGCGGGAGACGAACGA-3’ | 5’-CGCCAGCATCACCAACC-3’ | 189bp |
| *Sycn* | 5’-CGGGCTGGTCTAACACT-3’ | 5’-GGTCATCTTGCCTCCTG-3’ | 268bp |
| *0610005C13Rik* | 5’-CTTAGACGGGACAACGA-3’ | 5’-GAAGGTCAGCAGGGATG-3’ | 198bp |
| *Orc2* | 5’-TGTGCTTTATGGCTTGGGGT-3’ | 5’-CCCGCTGATCCAGAACACTT-3’ | 188bp |
| *Orc5* | 5’-CCTCCAGAGTATTCAGCG-3’ | 5’-CAGTTTGCGAGTATCACG-3’ | 174bp |
| *Cxcr2* | 5’-CTTCCAGTTCAACCAGCCCT-3’ | 5’-CTCTGAGTGGCATGGGACAG-3’ | 237bp |
| *Pacsin3* | 5’-TGTCTCTCTTTTCCCACAGGAG-3’ | 5’-GCCAGCCTCAAACTACATTCTC-3’ | 207bp |
| *Prkcz* | 5’-TAAGATAAAGAGCCCGCAGATG-3’ | 5’-CGAACCAGTAACCAGCAAGTC-3’ | 187bp |
| *Tnfsf9* | 5’-GTCCAACATTCACAAACACAGG-3’ | 5’-ATAAGCCCTCAGACCCACACT-3’ | 209bp |
| *Cxcl5* | 5’-GTTCCATCTCGCCATTCATG-3’ | 5-GCGGCTATGACTGAGGAAGG-3’ | 231bp |
| *Cxcr5* | 5’-ACATCAGACAGTGACCAGCC-3’ | 5’-CTGTAGGCCACAGGCATGAA-3’ | 223bp |
| *Gapdh* | 5’-AGGTCGGTGTGAACGGATTTG-3’ | 5’-TGTAGACCATGTAGTTGAGGTA-3’ | 129bp |
